# ^18^F-FDG uptake in the stomach on screening PET/CT: value for predicting *Helicobacter pylori* infection and chronic atrophic gastritis

**DOI:** 10.1186/s12880-016-0161-9

**Published:** 2016-10-18

**Authors:** Shigeki Kobayashi, Mayumi Ogura, Naohisa Suzawa, Noriyuki Horiki, Masaki Katsurahara, Toru Ogura, Hajime Sakuma

**Affiliations:** 1 Center for Preventive Medicine, Mie University Hospital, 2-174 Edobashi, Tsu, Mie Pref. 514-8507 Japan; 2 Department of Radiology, Mie University Hospital, 2-174 Edobashi, Tsu, Mie Pref. 514-8507 Japan; 3 Department of Gastroenterology and Hepatology, Mie University Hospital, 2-174 Edobashi, Tsu, Mie Pref. 514-8507 Japan; 4 Clinical Research Support Center, Mie University Hospital, 2-174 Edobashi, Tsu, Mie Pref. 514-8507 Japan

**Keywords:** ^18^F-FDG PET/CT, *Helicobacter pylori* infection, Chronic atrophic gastritis

## Abstract

**Background:**

The aim of this study was to determine the value of ^18^F-FDG uptake on screening PET/CT images for the prediction of *Helicobacter pylori* (*H. pylori*) infection and chronic atrophic gastritis.

**Methods:**

Among subjects who underwent ^18^F-FDG PET/CT for cancer screening from April 2005 to November 2015, PET/CT images were analyzed in 88 subjects who had gastrointestinal fiberscopy within 6 months. The volumes of interest (VOIs) were placed in the fornix, corpus and antrum of the stomach to determine maximal standardized uptake value (SUVmax) and mean SUV (SUVmean). Receiver operating characteristic curve (ROC) analysis was performed to determine the diagnostic performance of SUV indicators in predicting *H. pylori* infection and chronic atrophic gastritis.

**Results:**

SUV indicators of the stomach were significantly higher in subjects with *H. pylori* infection than those without (from *P* < 0.001 to *P* < 0.05). ROC analysis revealed that SUVmean had the highest performance in predicting *H. pylori* infection (AUC 0.807) and chronic atrophic gastritis (AUC 0.784). SUVmean exhibited the sensitivity of 86.5 % and the specificity of 70.6 % in predicting *H. pylori* infection, and the sensitivity of 75.0 % and 78.6 % in predicting chronic atrophic gastritis.

**Conclusion:**

Assessment of ^18^F-FDG uptake in the stomach reflecting active inflammation is useful in predicting patients with *H. pylori* infection and subsequent chronic atrophic gastritis which is closely associated with the risk of gastric neoplasms.

## Background

*Helicobacter pylori* (*H. pylori*) infection is strongly related with many gastroduodenal diseases including peptic ulcer diseases, chronic atrophic gastritis, mucosa associated lymphoid tissue (MALT) lymphoma and gastric cancer [1, 2]. In particular, gastric cancer is the third most common of all cancers among males and the fifth most common among females. Once infection of *H. pylori* is established, it usually lasts for life and exhibits carcinogenicity which induces gastric cancer through chronic atrophic gastritis [3].

^18^F-FDG PET/CT is widely used in cancer staging and cancer screening. However, previous studies demonstrated that the sensitivity of ^18^F-FDG-PET in screening gastric cancer in asymptomatic subjects was limited, ranging from 10 % to 38 % [4, 5]. The main difficulty in ^18^F-FDG-PET diagnosis of gastric cancer is attributed to physiological uptake of ^18^F-FDG in the stomach [6–10]. In addition to the abnormal ^18^F-FDG uptake associated to malignant tumors, physiological or inflammation related uptakes are seen on ^18^F-FDG PET images. Takahashi et al [11] evaluated the pattern of ^18^F-FDG uptake in the stomach in association with endoscopic findings of the gastric mucosa in 272 cases and found that accumulation pattern of ^18^F-FDG largely corresponds to the presence of mucosal inflammation. Although semi-quantitative evaluation of ^18^F-FDG uptake using standardized uptake values (SUVs) in the stomach has been used for assessing MALT lymphoma [12], differentiating malignant and benign gastric diseases [13] and predicting the prognosis of gastric carcinoma [14], the value of SUV measurement of FDG uptake for detecting *H. pylori* infection and subsequent chronic atrophic gastritis has not been well established. Lin et al [15] found a significant positive correlation between SUVs of ^18^F-FDG in the stomach and the values of C-13 urea breath test which is the most commonly used noninvasive test for *H. pylori*. However, the number of the subjects was limited (*n* = 16) and endoscopic examination was not performed in their study.

Consequently, the aim of this study was to investigate the value of semi-quantitative assessment of ^18^FDG uptake in the stomach with SUV for predicting *H. pylori* infection and chronic gastritis in subjects who underwent ^18^F-FDG PET/CT for cancer screening.

## Methods

### Subjects

Medical records of subjects who underwent ^18^F-FDG PET/CT for cancer screening between April 2005 and November 2015 were retrospectively investigated. Among them, 88 subjects underwent gastrointestinal fiberscopy within 6 months of the PET/CT study. The reasons for gastrointestinal fiberscopy were increased uptake of ^18^F-FDG in the stomach (55 subjects), previous history of peptic ulcer (5 subjects), familial history of peptic ulcer or gastric cancer (4 subjects), or request by the examinee (32 subjects). None of these 88 subjects had a previous history of gastric cancer or MALT lymphoma. The presence or absence of *H. pylori* infection, as well as the diagnosis of chronic atrophic gastritis, were determined in the medical records in all 88 subjects who underwent gastrointestinal fiberscopy. Thus, ^18^F-FDG PET/CT images were evaluated in these 88 subjects. This retrospective study was approved by the institutional review board of Mie University Hospital (study no. 2989) and was conducted in accordance with the Declaration of Helsinki and Good Clinical Practice. Informed consent was waived for this retrospective study.

### PET/CT imaging

All subjects fasted for at least 6 h before PET/CT acquisitions. Prior to ^18^F-FDG injection, blood glucose levels were determined from capillary blood samples and were confirmed to be less than 150 mg/dl in all subjects. A 3.7-MBq/kg dose of ^18^F-FDG was injected intravenously in one arm. PET/CT was performed by using an Aquiduo PCA-700B scanner (Toshiba, Nasu, Japan) or Discovery PET/CT 690 scanner (GE, Milwaukee, WI). Images from the skull to the mid-thigh were acquired approximately 60 min after ^18^F-FDG injection, by employing 3-dimentional acquisitions in 7-9 bed positions with 2-min acquisition in each position. Subjects were placed supine with the arms alongside the body or lifted up to the skull and were allowed to breathe normally during PET acquisitions. CT images acquired in approximately ten seconds during a natural breath-holding were used for attenuation correlation and generation of fusion images. Attenuation-corrected PET images with co-registered CT data were reviewed.

### PET/CT image analysis

**Fig. 1:**
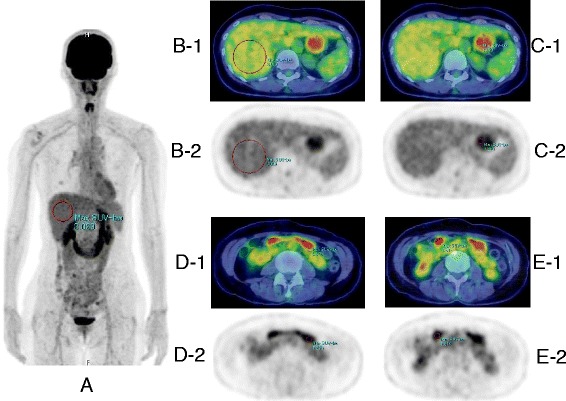
The methods for measuring ^18^F-FDG uptake of the stomach and the liver. VOIs of 3D sphere were placed at the fornix, corpus and antrum of the stomach and the liver in each subject. SUVmax (shown as Max SUV-bw on MIP, fusion images and PET images) and SUVmean were determined in each VOI in the stomach, and SUVmean liver was determined in liver VOI. **a** A MIP image of a subject with *H. pylori* infection, VOI was placed to avoid the area just blow the diaphragm for preventing the motion blurring artifact. The VOIs were placed by monitoring both PET/CT fusion images and PET images. **b**-1, 2 VOI of the liver. **c**1, 2 VOI of gastric fornix. **d**-1, 2 VOI of gastric corpus. **e**-1, 2 VOI of gastric antrum

^18^F-FDG uptake in the stomach was measured semi-quantitatively by placing volumes of interest (VOIs) at the fornix, corpus and antrum of the stomach as well as in the liver by consensus of two observers. The VOIs were 3D spheres and the size of VOIs were 5 mm in diameter for the stomach, and 30 mm in diameter for the liver. The VOI for the stomach was carefully placed in the gastric wall by monitoring both PET-CT fusion images and PET images. VOI for the liver was placed to avoid the region just blow the diaphragm for preventing the motion blurring artifact. For each VOI, maximal SUV (SUVmax) and mean SUV (SUVmean) were recorded (Fig. 1). ROC analysis was performed for several different SUV indicators. Maximum SUVmax and mean SUVmax were the maximum and mean values of the SUVmax measured at the fornix, corpus and antrum. Maximum SUVmean and mean SUVmean were the maximum and mean values of the SUVmean measured at the fornix, corpus and antrum. In addition, maximum SUVmax / SUVmean liver, mean SUVmax /SUVmean liver, maximum SUVmean / SUVmean liver and mean SUVmean / SUVmean liver were determined to evaluate the diagnostic performance of these indicators in predicting *H pylori* infection and chronic atrophic gastritis.

### Statistical analysis

SPSS version 22.0 software (IBM Japan, Tokyo, Japan) was used for statistical analyses. We determined whether statistically significance difference was observed in SUVs of the stomach between those with and without *H. pylori*, and those with and without chronic atrophic gastritis. The sensitivity and specificity of SUV indicators in predicting *H. pylori* infection and chronic atrophic gastritis were calculated by using an optimal cut-off point on the ROC curve that has the minimum distance to the upper left corner (where sensitivity = 1 and specificity = 1). The statistically significance was evaluated by Mann-Whitney *U*-test or Wilcoxon rank sum test. All analysis were 2-sided, a P-value of less than 0.05 was considered statistically significant.

## Results

### Characteristics of subjects

Characteristics of the subjects including laboratory diagnosis by gastrointestinal fiberscopy are shown in Table 1. Diagnosis of *H. pylori* infection was made by a rapid urease test, a stool antigen test and an information of previous medical institution or prevention center. Three subjects who had chronic atrophic gastritis without *H. pylori* infection on medical records in previous medical institutions, were turned out to be H. pylori positive by further investigation in our hospital.

**Table 1:** Characteristics of the subjects

Age (y)	
Mean ± SD	58 ± 11
Range	34 − 79
Gender	Number (%)
Female	38 (43.2)
Male	50 (56.8)
*H. Pylori* infection	Number (%)
Positive	37 (42.0)
Negative	51 (58.0)
Chronic atrophic gastritis	Number (%)
Positive (*H. Pylori* positive)	37 (40.9)
(*H. Pylori* negative)	24 (27.3)
Negative	27 (31.8)
Neoplasms (finding on fiberscopy)	Number
Early gastric cancer (*H. Pylori* positive)	4
Gastric adenoma (*H. Pylori* positive)	2
MALT lymphoma (*H. Pylori* positive)	1
Other fiberscopic findings	3
Superficial gastritis (*H. Pylori* positive)	3
(*H. Pylori* negative)	2
Erosive gastritis (*H. Pylori* positive)	2
(*H. Pylori* negative)	3
Gastric ulcer scar (*H. Pylori* positive)	1
Duodenal ulcer scar (*H. Pylori* positive)	1
Erosion of E-C Junction (*H. Pylori* negative)	1
Reflux esophagitis (*H. Pylori* positive)	1
(*H. Pylori* negative)	1

### PET/CT image analysis

Table 2 summarizes the SUVmax and SUVmean of 18 F-FDG uptake at the fornix, corpus and antrum, as well as the maximum and the mean values of SUVmax and SUVmean at 3 regions in the stomach in associated with *H. pylori* infection. Table 3 summarizes these SUV indicators in associated with chronic atrophic gastritis. All of these SUV indicators in the stomach were significantly higher in patients with *H. pylori* infection than in those without *H. pylori* (*P* < 0.001). In addition, all of these SUV indicators were significantly higher in patients with chronic atrophic gastritis than in those without chronic atrophic gastritis (*P* < 0.001). It was also noted that the 18 F-FDG uptake of the fornix in the stomach was significantly higher than those in corpus and antrum, independent of the presence of *H. pylori* infection and chronic atrophic gastritis.

**Table 2:** The SUVs of 18 F-FDG in the stomach in associated with *Helicobacter pylori* infection

	*H. pylori*(+) N = 37	*H. pylori*(-) N = 51
SUVmax (mean ± SD)		
Fornix	4.01 ± 0.80	3.38 ± 0.97^++^
Corpus	3.70 ± 0.95^**^	2.71 ± 0.90^*+^
Antrum	3.58 ± 1.12^***^	2.68 ± 0.99^*+^
Maximum	4.36 ± 0.88	3.57 ± 1.01
Mean	3.76 ± 0.78	2.93 ± 0.80^+^
SUVmean (mean ± SD)		
Fornix	3.62 ± 0.71	3.06 ± 0.90^++^
Corpus	3.31 ± 0.87^***^	2.39 ± 0.83^*+^
Antrum	3.06 ± 0.96^**^	2.30 ± 0.87^*+^
Maximum	3.90 ± 0.74	3.19 ± 0.92^+^
Mean	3.33 ± 0.67	2.58 ± 0.73^+^

Significant difference between each region and fornix at same group (*P < 0.001, **P < 0.01, ***P < 0.05)

Significant difference between H. Pylori (+) and H. Pylori (-) at same region (+*P* < 0.001, ++*P* < 0.01, +++*P* < 0.05)

**Table 3:** The SUVs of 18 F-FDG in the stomach in associated with chronic gastritis

	Chr Gastritis(+) N = 60	Chr Gastritis(-) N = 28
SUVmax (mean ± SD)		
Fornix	3.86 ± 0.91	3.20 ± 0.92^+^
Corpus	3.39 ± 1.00^*^	2.57 ± 0.92^*+^
Antrum	3.37 ± 1.19^**^	2.39 ± 0.67^*+^
Maximum	4.12 ± 0.97	3.42 ± 1.02^++^
Mean	3.54 ± 0.89	2.72 ± 0.62^+^
SUVmean (mean ± SD)		
Fornix	3.49 ± 0.85	2.88 ± 0.80^+++^
Corpus	3.02 ± 0.94^*^	2.25 ± 0.81^*+^
Antrum	2.88 ± 1.01^*^	2.06 ± 0.65^*+^
Maximum	3.69 ± 0.88	3.06 ± 0.90^++^
Mean	3.13 ± 0.79	2.40 ± 0.55^+^

Significant difference between each region and fornix at same group (*P < 0.001, **P < 0.01)

Significant difference between Chr Gastritis (+) and Chr Gastritis (-) at same region (+ *P* < 0.001, ++ *P* <0.01, +++ *P* < 0.05)

### Diagnostic performance by ROC analysis

**Fig. 2:**
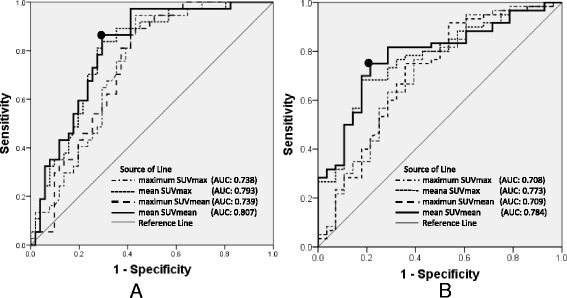
ROC curves for SUV indicators. **a** ROC curves for predicting *H. pylori* infection. **b** ROC curves for predicting chronic atrophic gastritis. Among these SUV indicators, the highest diagnostic performance was achieved with the mean SUVmean in the fornix, corpus and antrum for predicting *H. pylori* infection as well as for predicting chronic atrophic gastritis

Figure 2 shows ROC curves for SUV indicators in predicting *H. pylori* infection and chronic atrophic gastritis. In Table 4, the area under ROC curves, the optimal cut-off values, the sensitivities and specificities in predicting *H. pylori* infection and chronic atrophic gastritis are presented. All of SUV indicators demonstrated good diagnostic performance for the prediction of *H. pylori* infection and chronic atrophic gastritis. Among these 4 SUV indicators, mean SUVmean exhibited the highest area under ROC curves for predicting *H. pylori* infection (0.807, 95%CI 0.715 – 0.898) and for chronic gastritis (0.784, 95 % CI 0.684 – 0.884). As shown in Table 5, normalization of these SUV indicators in the stomach by the liver SUV did not improve the area under ROC curves for the diagnosis of *H. pylori* infection. For predicting chronic atrophic gastritis, normalization of SUV indicators by the liver SUV slightly improve the area under ROC curves, with the highest area under ROC curve of 0.793 (95 % CI 0.686 – 0.900) by mean SUVmax. However, the amount of improvement with normalization by the liver SUV was quite limited.

**Table 4:** Diagnostic performance of SUVs for *H. pylori* infection

Predictive Indicators	AUC	Cut-off	Sensitivity	Specificity	95 % CI of AUC	*P* value
Maximun SUVmax	0.738	3.66	81.1 %	60.8 %	0.635 – 0.841	<0.001
Mean SUVmax	0.793	3.11	81.1 %	72.5 %	0.699 – 0.887	< 0.001
Maximun SUVmean	0.739	3.30	81.1 %	62.7 %	0.636 – 0.841	< 0.001
Mean SUVmean	0.807	2.66	86.5 %	70.6 %	0.715 – 0.898	< 0.001
Diagnostic performance of SUVs for chronic atrophic gastritis						
Maximun SUVmax	0.708	3.42	76.7 %	60.7 %	0.585 – 0.831	0.02
Mean SUVmax	0.773	2.86	76.7 %	67.9 %	0.671 – 0.875	< 0.001
Maximun SUVmean	0.709	3.15	75.0 %	64.3 %	0.585 – 0.833	0.02
Mean SUVmean	0.784	2.57	75.0 %	78.6 %	0.684 – 0.884	< 0.001

**Table 5:** Diagnostic performance of SUVs normalized by SUV in the liver for *H. Pylori* infection

Predictive Indicators	AUC	Cut-off	Sensitivity	Specificity	95 % CI of AUC	*P* value
Maximun SUVmax	0.739	1.64	64.9 %	72.5 %	0.637 – 0.841	<0.001
Mean SUVmax	0.796	1.31	81.1 %	74.5 %	0.700 – 0.892	<0.001
Maximum SUVmean	0.738	1.37	83.8 %	60.8 %	0.635 – 0.841	<0.001
Mean SUVmean	0.791	1.15	81.1 %	72.5 %	0.695 – 0.887	<0.001
Diagnostic performance of SUVs normalized by SUV in the liver for chronic atrophic gastritis						
Maximun SUVmax	0.721	1.44	73.3 %	60.7 %	0.596 – 0.847	0.01
Mean SUVmax	0.793	1.25	76.7 %	78.6 %	0.686 – 0.900	<0.001
Maximum SUVmean	0.711	1.25	83.3 %	60.7 %	0.583 – 0.838	0.02
Mean SUVmean	0.790	1.09	78.3 %	75.0 %	0.682 – 0.897	<0.001

**Fig. 3:**
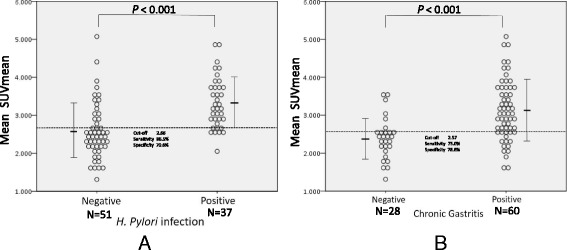
Distribution of mean SUVmean values. **a** Dot plots for mean SUVmean values in subjects with and without *H. pylori* infection. **b** Dot plots for mean SUVmean values in subjects with and without chronic atrophic gastritis. Statistical significant difference for the mean SUVmean values was observed between subjects with and without *H. pylori* infection (*p* < 0.001) and between subjects with and without chronic gastritis (*p* < 0.001)

Dot plots of mean SUVmean in subjects with and without *H. pylori* infection and in those with and without chronic gastritis were shown on Fig. 3. The sensitivity and specificity of mean SUVmean were 86.5 % and 70.6 % for *H. pylori* infection (optimal cut-off value of 2.66), and 75.0 % and 78.6 % for chronic gastritis (optimal cut-off value of 2.57), respectively.

### Gastric neoplasms found by GIF

Among the 88 subjects, seven neoplasms were found on gastrointestinal fiberscopy, including four early gastric cancers, two gastric adenomas and a MALT lymphoma. In four gastric cancer and two gastric adenomas, no focal increase in ^18^F-FDG uptake corresponding to tumors was observed, while *H. pylori* was positive in these cases. In a patient with MALT lymphoma, the antibody test of *H. pylori* was negative and increased focal ^18^F-FDG uptake at gastric corpus was detected which corresponded to MALT lymphoma proven by gastrointestinal fiberscopy. The infection of *H. pylori* was also demonstrated by histologic specimen taken by fiberscopy.

## Discussion

In the current study, we investigated the value of 18 F-FDG uptake measured by SUV on screening PET/CT images for the prediction of *H. pylori* infection and chronic atrophic gastritis determined by gastrointestinal fiberscopy. The major findings in this study were [1] The SUV of 18 F-FDG uptake in the stomach was significantly elevated in patients with *H. pylori* infection and in those with chronic atrophic gastritis [2]; 18 F-FDG uptake of the fornix in the stomach was significantly higher than those in corpus and antrum regardless of *H. pylori* infection and chronic atrophic gastritis [3]; mean SUVmean showed the highest area under ROC curves for predicting *H. pylori* infection (0.807) and chronic atrophic gastritis (0.784), and is useful for identifying patients who require gastrointestinal fiberscopy. Normalization of stomach SUVs by liver SUV provided minimal differences in the diagnostic performance and is not considered to be necessary.

### Accumulation of ^18^F-FDG in the stomach

Pattern of accumulation of ^18^F-FDG in the stomach and its associated with endoscopic findings of the gastric mucosa and *H. pylori* infection were previously investigated by Takahashi et al [11] by using a visual assessment of ^18^F-FDG PET image. They classified ^18^F-FDG uptake in the stomach into three groups (A: localized accumulation in the fornix, B: diffuse accumulation throughout the entire stomach, C: no accumulation). They found that *H. pylori* infections were more frequent in Groups A and B than in Group C, concluding that accumulation of ^18^F-FDG in the stomach suggests a high probability of inflammatory changes to the gastric mucosa, forming a background for the development of cancer or malignant lymphoma. In our current study, we used a more objective approach by measuring SUVs of ^18^F-FDG in the fornix, corpus and antrum. Consistent with previous report [8, 11], we found that ^18^F-FDG uptake of the fornix was significantly higher than corpus and antrum. In addition, SUV of ^18^F-FDG in the fornix was significantly higher than those in corpus and antrum, not only in the subjects with *H. pylori* infection and chronic atrophic gastritis, but also in those without *H. pylori* infection or chronic atrophic gastritis, suggesting that high ^18^F-FDG uptake in an oral side of the stomach is physiological. We also noticed that *H. pylori* infection and chronic atrophic gastritis are associated with elevated SUVs in all gastric regions including the fornix, corpus and antrum. This indicates that *H. pylori* infection causes increased ^18^F-FDG uptake reflecting active inflammation throughout the entire stomach.

### Detection of gastric neoplasms by ^18^F-FDG PET/CT

For the assessment of patients with advanced gastric cancer, ^18^F-FDG PET/CT has been shown to be useful in detecting nodal metastasis and distant metastasis, and in predicting prognosis [16–20]. However, ^18^F-FDG PET/CT is not useful for screening gastric cancers [5, 11, 21, 22]. Shoda et al. studies 2861 asymptomatic subjects and found that the sensitivity of ^18^F-FDG PET for gastric cancer was as low as 10 % [3]. Consequently the use of gastrointestinal fiberscopy is considered more appropriate in screening gastric cancer.

### Clinical implications

Our results demonstrated that semi-quantitative assessment of ^18^F-FDG uptake with SUV has high diagnostic accuracy in predicting *H. pylori* infection and chronic atrophic gastritis. As previously mentioned, *H. pylori* infection and subsequent chronic atrophic gastritis lead to increased risk of gastric cancer formation. Inflammatory change in the gastric mucosa caused by *H. pylori* forms a background for the development of gastric cancer or malignant lymphoma. In a Japanese cohort study, the population attributable fraction (PAF) of *H. pylori* infection for gastric cancer incidence (i.e. the fraction of gastric cancer incident cases that is attributable to *H. pylori* infection) was estimated to be 84 % [3]. Despite the declined prevalence of *H. pylori* infection for the past 30 years, gastric cancer is the second most frequent cause of cancer death in both males and females in Japan, and the most frequent cancer in males and the second most frequent cancer in females [23]. Therefore, gastrointestinal fiberscopy should be strongly recommended for subjects with increased ^18^F-FDG uptake in the stomach. According to the results in this study, high area under ROC of 0.807 and high sensitivity of 86.5 % can be achieved when mean SUVmean values of > 2.66 was used as a threshold.

### Limitations

Several limitations must be acknowledged in this study. First, this is a single-center study with a limited number of subjects, and there is a selection bias for subjects who underwent gastrointestinal fiberscopy. Second, the degree of chronic atrophic gastritis was not evaluated in current study, because the laboratory diagnosis by gastrointestinal fiberscopy was qualitative and operator-dependent. Third, CT images were acquired during natural breath-holding while PET images were obtained during free-breathing. This may result in misregistration artifact and alteration in SUV. Forth, SUV of ^18^F-FDG uptake in the stomach was not compared with the gastrointestinal fiberscopy findings in detail. Further investigation by prospective multi-center study using both PET-CT and gastrointestinal fiberscopy is necessary to determine the value of ^18^F-FDG PET in early detection and prevention of gastric cancer.

## Conclusion

Uptake of ^18^F-FDG in the stomach reflecting active inflammation is strongly associated with *H. pylori* infection and subsequent chronic atrophic gastritis. Subjects demonstrating increased SUV of ^18^F-FDG uptake in the stomach should be recognized as patients with high likelihood *H. pylori* infection and at increased risk of gastric neoplasms. Gastrointestinal fiberscopy should be recommended in these subjects.

## Abbreviations

^18^F-FDG PET/CT: [^18^F] fluoro-2-deoxy-D-glucose positron emission tomography / computed tomography
AUC: Area under the curve
CI: Confidence interval
FDG: Fluoro-2-deoxy-D-glucose
MALT: Mucosa associated lymphoid tissue
PET/CT: Positron emission tomography / computed tomography
ROC: Receiver operating characteristic curve
SUV: Standardized uptake value
SUVmax: Maximal standardized uptake value
SUVmean: Mean standardized uptake value
